# Identification of Glucose Transporters in *Aspergillus nidulans*

**DOI:** 10.1371/journal.pone.0081412

**Published:** 2013-11-25

**Authors:** Thaila Fernanda dos Reis, João Filipe Menino, Vinícius Leite Pedro Bom, Neil Andrew Brown, Ana Cristina Colabardini, Marcela Savoldi, Maria Helena S. Goldman, Fernando Rodrigues, Gustavo Henrique Goldman

**Affiliations:** 1 Laboratório Nacional de Ciência e Tecnologia do Bioetanol – CTBE, Campinas, São Paulo, Brazil; 2 Faculdade de Ciências Farmacêuticas de Ribeirão Preto, Universidade de São Paulo, São Paulo, Brazil; 3 Faculdade de Filosofia, Ciências e Letras de Ribeirão Preto, Universidade de São Paulo, São Paulo, Brazil; 4 Life and Health Sciences Research Institute (ICVS), School of Health Sciences, University of Minho, Braga, Portugal; Texas A&M University, United States of America

## Abstract

To characterize the mechanisms involved in glucose transport, in the filamentous fungus *Aspergillus nidulans*, we have identified four glucose transporter encoding genes *hxtB-E*. We evaluated the ability of *hxtB-E* to functionally complement the Saccharomyces cerevisiae EBY.VW4000 strain that is unable to grow on glucose, fructose, mannose or galactose as single carbon source. In *S. cerevisiae* HxtB-E were targeted to the plasma membrane. The expression of HxtB, HxtC and HxtE was able to restore growth on glucose, fructose, mannose or galactose, indicating that these transporters accept multiple sugars as a substrate through an energy dependent process. A tenfold excess of unlabeled maltose, galactose, fructose, and mannose were able to inhibit glucose uptake to different levels (50 to 80 %) in these s. *cerevisiae* complemented strains. Moreover, experiments with cyanide-*m*-chlorophenylhydrazone (CCCP), strongly suggest that *hxtB*, *-C*, and –*E* mediate glucose transport via active proton symport. The *A. nidulans ΔhxtB*, *ΔhxtC* or *ΔhxtE* null mutants showed ~2.5-fold reduction in the affinity for glucose, while *ΔhxtB* and -C also showed a 2-fold reduction in the capacity for glucose uptake. The *ΔhxtD* mutant had a 7.8-fold reduction in affinity, but a 3-fold increase in the capacity for glucose uptake. However, only the *ΔhxtB* mutant strain showed a detectable decreased rate of glucose consumption at low concentrations and an increased resistance to 2-deoxyglucose.

## Introduction

Glucose represents the main source of carbon and energy for most heterotrophic organisms, in turn influencing the regulation of cell growth, metabolism and development [1]. When glucose is available, the synthesis of enzymes specific for the use of alternative, less preferred, carbon sources are repressed by a mechanism termed carbon catabolite repression (CCR) [2]. The action of the orthologous transcriptional repressors Mig1 and CreA/1, in *Saccharomyces cerevisiae* and filamentous fungi respectively, is central to CCR [3–6]. Subsequently, the sensing of extracellular and intracellular glucose, in addition to glucose transport, which occurs via facilitated diffusion [7] represent key events in the regulation of carbohydrate metabolism. 

Budding yeast *S. cerevisiae* has widely been used as a model system for the study of hexose sensing and transport [1,8-12]. In *S. cerevisiae*, extracellular glucose is sensed by two specific transmembrane proteins that act as sensors, Rgt2 and Snf3, which demonstrate similarity to hexose transporters (Hxt proteins). However, these sensor proteins are unable to transport glucose and have unusually long C-terminal tails (around 200 amino acids) that are predicted to reside in the cytoplasm [13] and are necessary for the sensing mechanisms [14-16]. In the absence of extracellular glucose, a transcriptional repressor complex, comprised of Rgt1, Std1 and Mth1, is bound to the promoter regions of *HXT* genes inhibiting transcription [17]. Then when Snf3 and Rgt2 detect extracellular glucose, the Std1 and Mth1 co-repressors are phosphorylated by the Yck1 and Yck2 kinases [18] and targeted to the SCFGrr1 E2/E3 ubiquitin complex for degradation [19-21]. This process results in the protein kinase A (PKA) mediated hyperphosphorylation of Rgt1, releasing it from the promoter regions of *HXT* genes, allowing their transcription [22]. Interestingly, the Snf3 and Rgt2 sensors induce the transcription of specific *HXT* genes. 

Hxt proteins form part of the sugarporter family within the Major Facilitator Superfamily (MSF) group [23]. In *S. cerevisiae*, twenty proteins have been classified as hexose transport proteins, with different Hxt proteins being transcriptionally induced depending upon the concentration of glucose available. Individual transporters have specific functions, since they all possess different substrate affinities or specificities such as (i) low-affinity Hxt1p and Hxt3p [Km(glucose) 100 mM]; (ii) moderate to low affinity Hxt2p and Hxt4p [Km(glucose), 10 mM]; and (iii) high affinity Hxt6p and Hxt7p [Km(glucose) 1–2 mM] [24]. Differences in individual *HXT* gene expression are not only dependent upon the concentration of available glucose but also upon osmotic pressure, starvation, and the physiological state of the cell [1,15,16,25-32]. 

Although considerable progress has been made in the understanding of how *S. cerevisiae* senses glucose, the equivalent knowledge of how filamentous fungi sense the presence of, and uptake, sugar is lacking. Only a single putative glucose sensor, *rco-3*, has been described in *Neurospora crassa* [33]. In addition, only a few glucose transporters have been characterized, such as the high affinity glucose transporters in *Amanita muscaria AmMst1*, in *Uromyces fabae HXT1*, in *Tuber borchii TBHX* and *N. crassa hgt-1* [34-39]. In the hemibiotrophic plant pathogen *Colletotrichum graminicola* several low and high affinity glucose transporters have been characterized and demonstrated infection phase specific regulation [40]. In Aspergilli, the *A. niger mstA* gene was shown to encode a high affinity glucose transporter [41] while the *A. nidulans hxtA* and *mstE* genes were characterized as a high affinity hexose transporter and a low affinity glucose transporter, respectively [42,43]. Recently, a high affinity glucose transporter, Hxt, was identified in *Fusarium oxysporium* that is able to transport glucose and xylose [44].

In order to characterize the mechanisms involved with glucose transport in the filamentous fungus *A. nidulans*, we have identified and characterized four putative glucose transporter homologues. To characterize their kinetic properties, we have expressed each homologue in a S. *cerevisiae* strain that cannot grow on D-glucose as a single carbon source. *A. nidulans* null mutants for these genes were analyzed for their ability to transport glucose. Using the aforementioned approaches, we were able to classify these genes as glucose transporters. 

## Results

### Identification of glucose transporter homologues in *A. nidulans*

**Figure 1:**
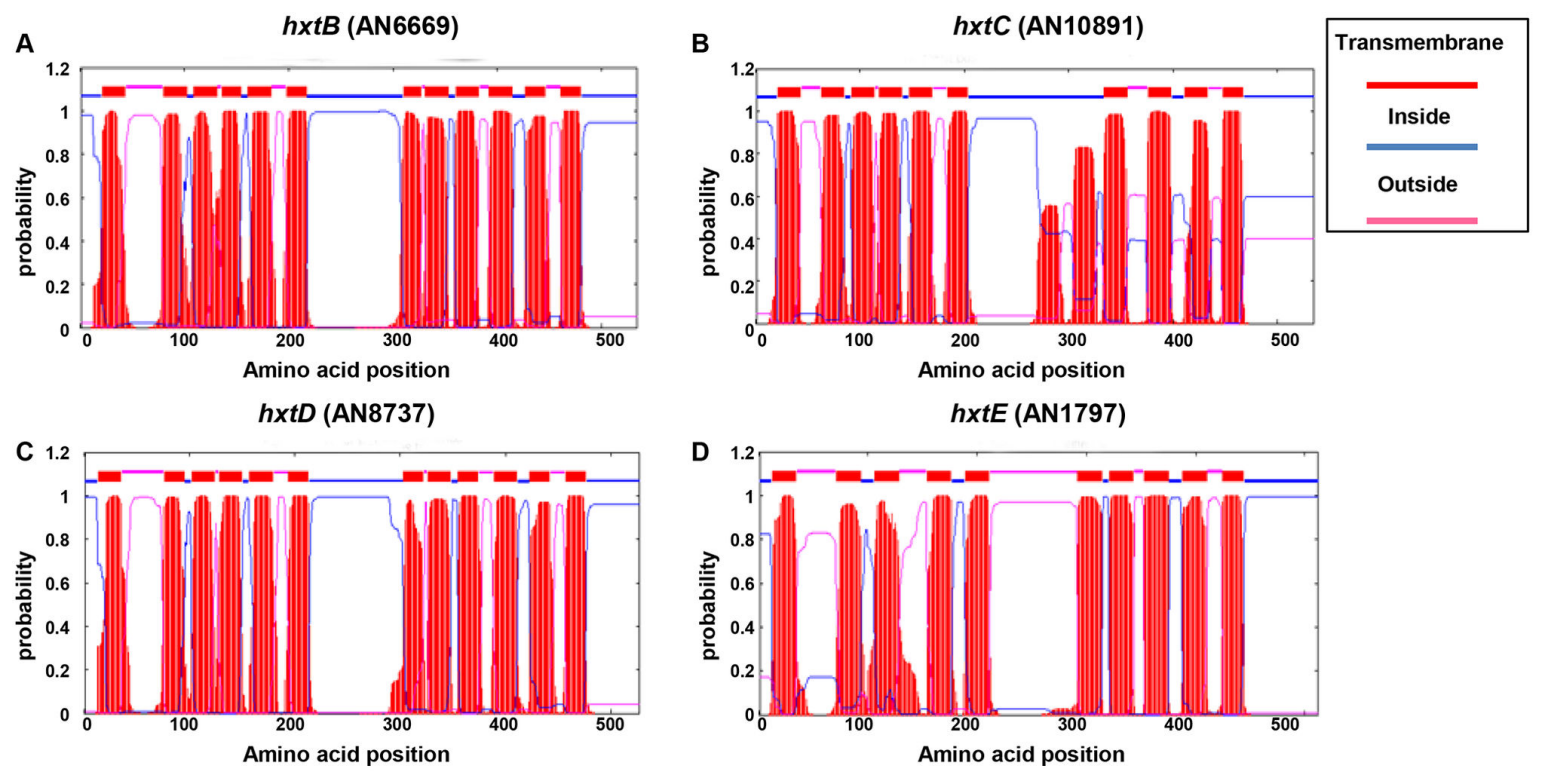
Transmembrane helices prediction for the *A. nidulans* HxtB-E transporters (predicted via TMHMM; http://www.cbs.dtu.dk/services/TMHMM/) and long C-terminal tails. The HxtB (A) and HxtD (C) contain 12 helices, while HxtC (B) and HxtE (D) contain 10 helices.

**Figure 2:**
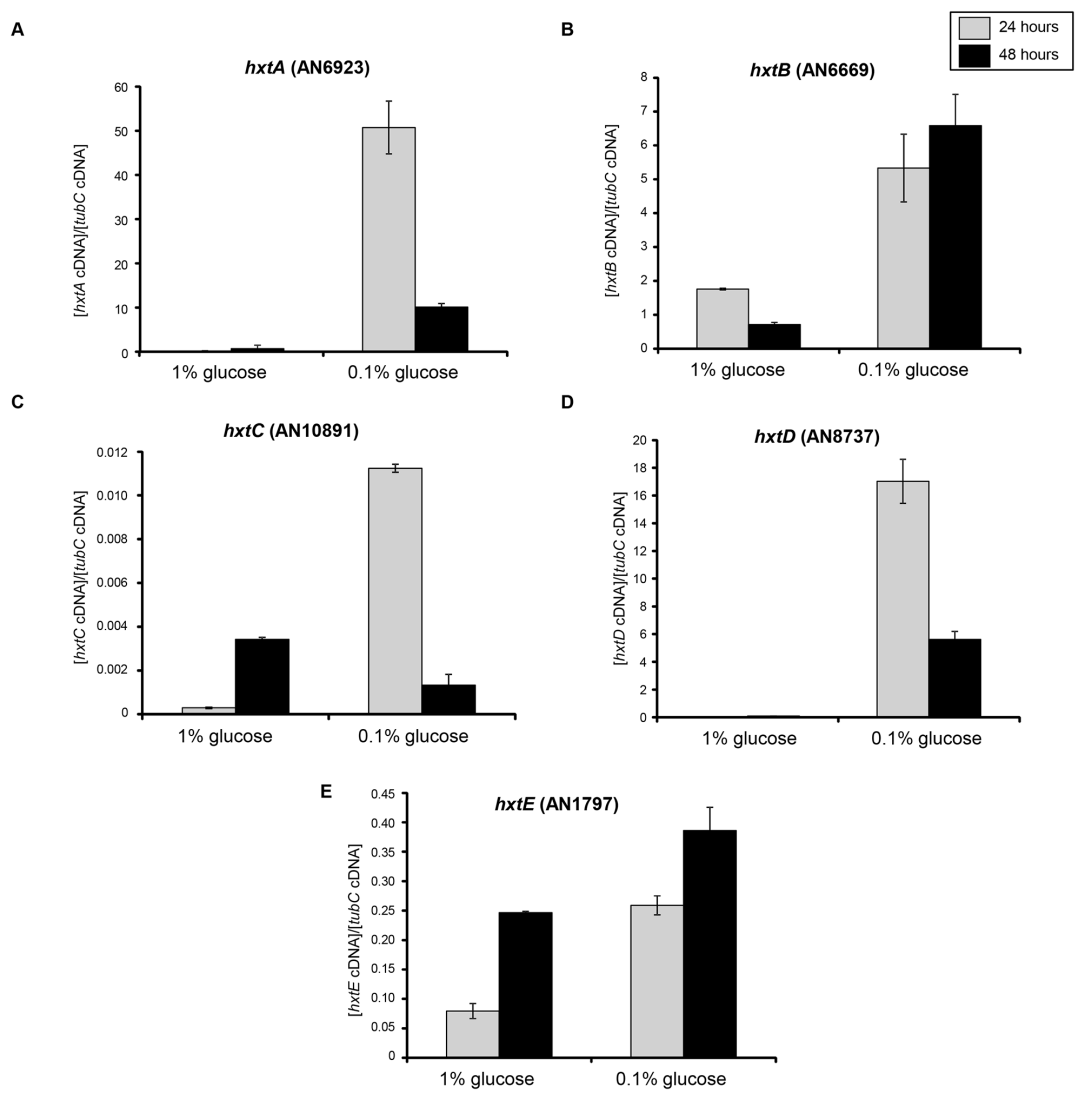
The *A. nidulans* *hxtA-E* mRNA accumulation levels during growth in 0.1 or 1 % glucose. The wild-type strain was grown for 24 or 48 hours in MM liquid medium supplemented with either 0.1 or 1 % glucose. Real-time qPCR for *hxtA-E* (A-E) genes.

 A BLASTp search of the *A. nidulans* genome (http://www.aspgd.org) using several genes from different fungal species that have been functionally identified as encoding glucose transporters [33-44] revealed four open reading frames as the best hits, with significant similarity to most of them (Table 1). The proteins of the four potential homologues, AN1797, AN10891, AN8737, and AN6669 (here named *hxtB-E*) were predicted to be from 527 to 535-amino acids in length and all belonged to the sugar porter subfamily of the Major Facilitator Superfamily (MFS). The HxtB and HxtD proteins contained 12 transmembrane segments (Figures 1A and C), while HxtC and HxtE contained only 10 helices (Figures 1B and D). All four Hxt transporters possessed a short C-terminal tail (Figure 1 A-D). Subsequently, the transcription of the four *hxt* genes when *A. nidulans* is grown in the presence of either 1 or 0.1 % glucose was confirmed via RT-qPCR (Figure 2). A putative high-affinity glucose transporter, *hxtA*, which has increased mRNA accumulation when *A. nidulans* is grown in the presence of low glucose concentrations or during carbon starvation was used as a control [42]. As previously described, *hxtA* showed higher mRNA accumulation at 0.1 % glucose (Figure 2A), while *hxtB*, *hxtC*, *hxtD*, and *hxtE* also showed higher levels of mRNA accumulation in 0.1 % than in 1.0 % glucose (Figures 2B- E). 

**Table 1:** *A. nidulans* putative glucose transporters identified as possible homologues of fungal glucose transporters.

**Genes**	**Species**	**AN1797 (*hxtB*)**		**AN10891 (*hxtC*)**		**AN8737 (*hxtD*)**		**AN6669 (*hxtE*)**	
		**Identity (%)**	**e-value**	**Identity (%)**	**e-value**	**Identity (%)**	**e-value**	**Identity (%)**	**e-value**
Rco3	*N. crassa*	49	0.0	94	1e-173	41	4e-125	42	6e-134
Hgt1	*N. crassa*	30	1e-64	0	0	29	4e-63	30	6e-69
Mst1	*A. muscaria*	51	2e-159	50	2e-175	47	3e-152	48	7e-156
Hxt1	*T. borchii*	59	0.0	60	0.0	47	1e-157	47	1e-152
Hxt1	*C. graminearum*	60	0.0	67	0.0	48	8e-158	47	1e-156
MstA	*A. niger*	80	0.0	0.0	0	81	0.0	47	3e-139
Hxt1	*U. fabae*	47	2e-149	50	4e-145	43	3e-133	43	3e-133
HxtA	*A. nidulans*	29	2e-62	28	2e-58	29	3e-63	28	2e-60
MstE	*A. nidulans*	33	2e-93	32	3e-90	32	2e-88	33	2e-88
Hxt1	*F. oxysporum*	29	6e-47	32	2e-47	28	3e-51	28	4e-52

**Figure 3:**
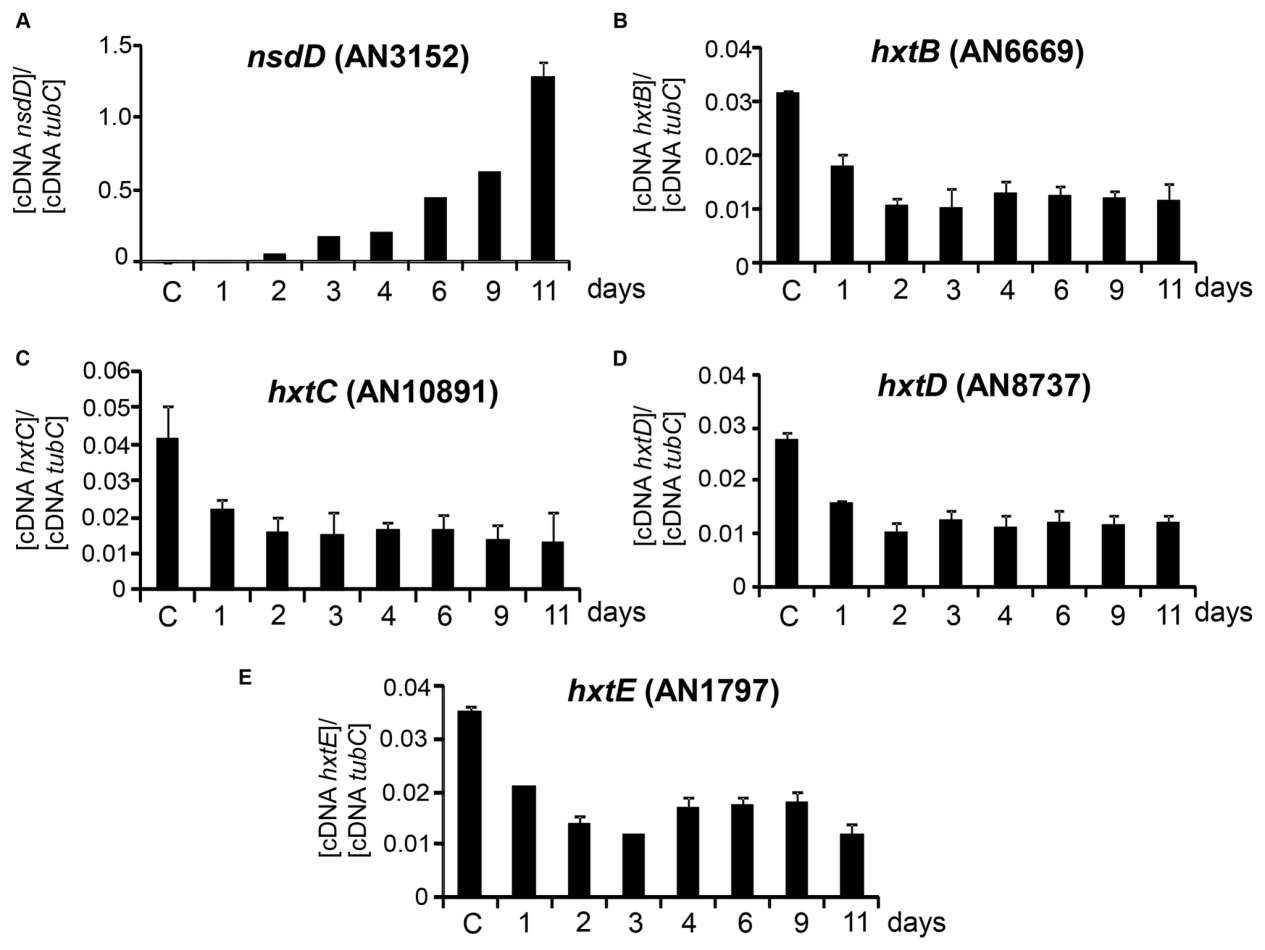
The *A. nidulans* *hxtA-E* mRNA accumulation levels during asexual and sexual development. Asexual spore development was synchronized by transferring a thin mycelial mat filtered from liquid culture (grown stationary at 37 °C for 24 hours; C=control) to an agar plate. To induce sexual development, we incubated the mycelia for 11 days (0–2 days: conidiophore development and asexual development; 2–11 days: cleistothecia development and sexual development; and 6–11 days: the presence of ascospores). Real-time qPCR for *hxtA-E* (A-E) genes.

To gain more insight into the function of the *hxtB-E* genes during *A. nidulans* sexual development, we examined their expression during the sexual cycle (Figure 3). First, asexual spore development was synchronized by transferring a thin mycelial mat filtered from liquid culture to an agar plate [45]. The exposure of cells to an air interphase induces development and conidiophores formation by 24–48 h. To induce sexual development, we incubated the mycelia for 11 days. By the third day, young cleistothecia could be observed. By the sixth and eleventh days, immature and mature ascospores could be detected respectively (data not shown). Total RNA was isolated at the different stages of sexual development and analyzed by real-time qPCR to determine transcript levels of *nsdD* and *hxtB-D* genes (Figure 3). The *nsdD* gene encodes a predicted GATA-type zinc-finger transcription factor required for sexual development [46]. As expected, the *nsdD* gene showed increased mRNA accumulation during sexual development (Figure 3A). The *hxtB-E* showed increased mRNA accumulation during vegetative growth (control), but they showed decreased mRNA accumulation in both asexual development (the first 48 h) and sexual development (Figure 3B-E).

### Characterization of the *hxtB-E* Genes in *S. cerevisiae*

**Figure 4:**
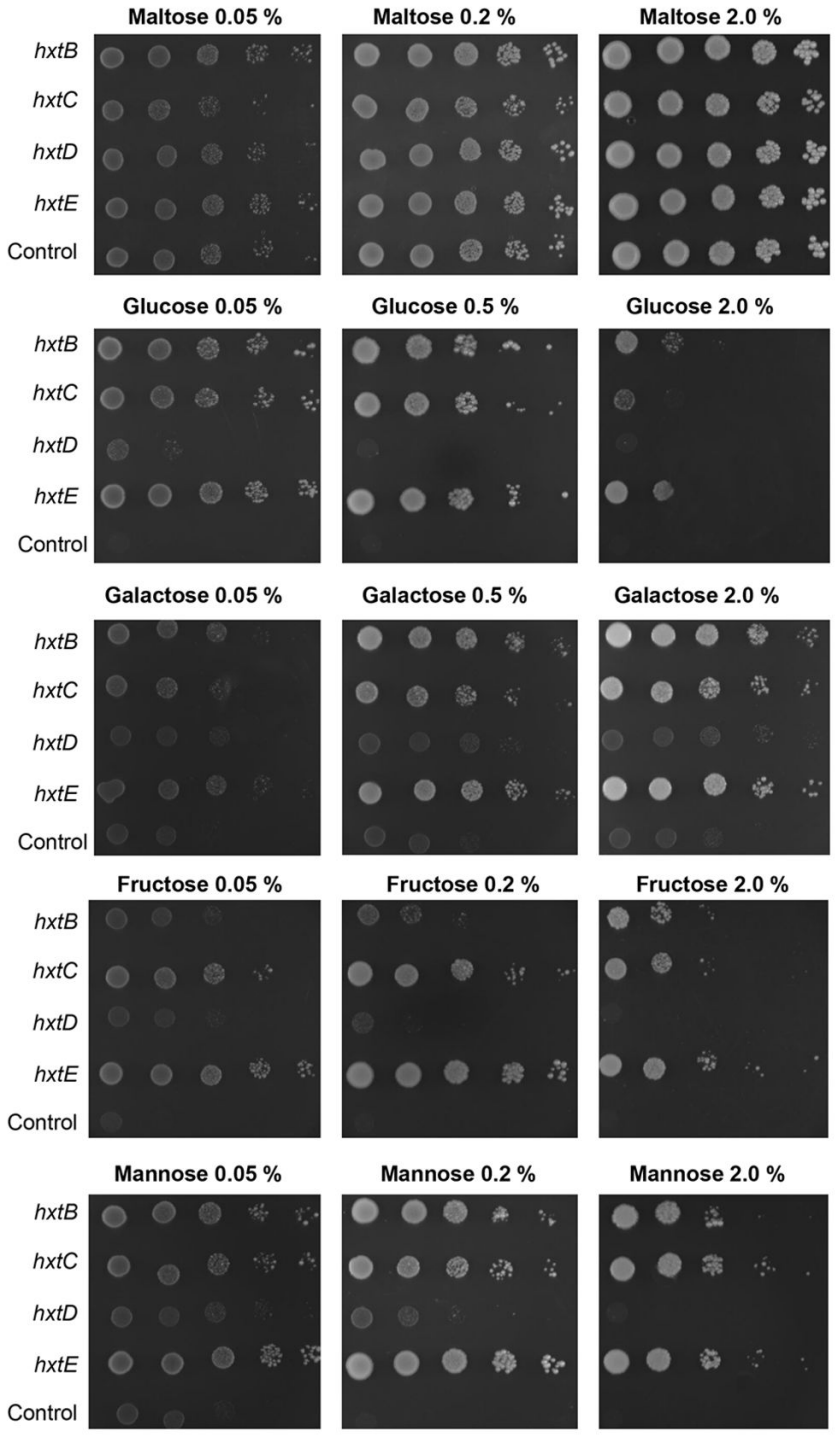
Comparative growth analyses of the *S. cerevisiae* cells expressing one of the four *hxtB-E* transporters. Tenfold dilutions (left to right) of *S. cerevisiae* cells (strain EBY.VW4000) expressing the indicated *hxt* cDNA or harbouring the empty expression vector were spotted on agar medium and incubated for 144 hour at 30 °C on plates containing the indicated carbon source.

**Figure 5:**
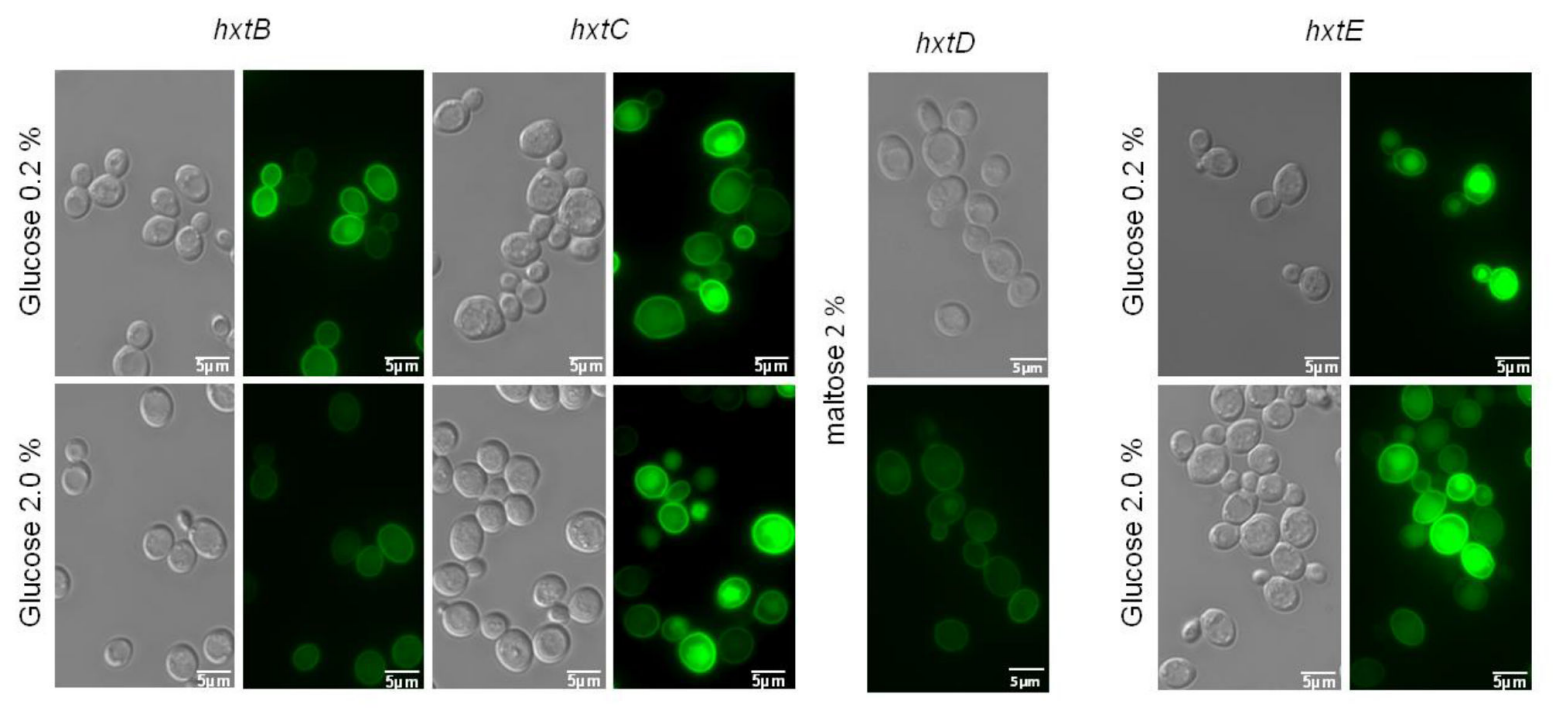
Subcellular localization of *hxtB-E* in *S. cerevisiae*. Subcellular localization of *hxtB-E* in *S. cerevisiae* grown in: 0.2% glucose, 2% glucose or 2% maltose, was determined by fluorescence microscopy. Scale bar, 5 μm.

 To show the functionality of the putative *A. nidulans* glucose transporter-encoding genes, we evaluated functional complementation of *hxtB-*E in the S. *cerevisiae* strain EBY.VW4000, which is unable to grow on glucose, fructose, mannose or galactose as the sole carbon source [47]. Subsequently, *hxtB-E* were cloned into the centromeric modified vector pRH195 under the control of the *HXT7* promoter and terminator. Transformants were selected in maltose liquid medium, and serial dilutions of logarithmically growing cells were spotted in onto YNB agar plates containing either one of the following carbon sources: glucose, fructose, mannose or galactose, at a range of different concentrations. Maltose was used as a positive control for growth and a transformant carrying the empty plasmid was used as negative control, where no growth was observed on medium containing sugars that do not sustain the EBY.VW4000 strain (Figure 4). The drop-out assay showed that the expression of HxtB, HxtC or HxtE was able to restore the growth of EBY.VW4000 on glucose, indicating that the corresponding genes encode glucose transporters (Figure 4). Moreover the strains expressing HxtB, HxtC or HxtE were also able to grow on fructose, mannose or galactose indicating that the encoded transporters accept multiple sugars as a substrate. However, their growth was inhibited at higher sugar concentrations, such as 2.0 % (Figure 4). The S. *cerevisiae* strain expressing *hxtD* was unable to grow on glucose or fructose and displayed very little growth on mannose or galactose (Figure 4). In *S. cerevisiae*, HxtB-E were confirmed to be targeted to the plasma membrane (Figure 5). Thus, the inability of *hxtD* to restore EBY.VW4000 growth on glucose cannot be explained by the incorrect targeting of the protein. 

**Figure 6:**
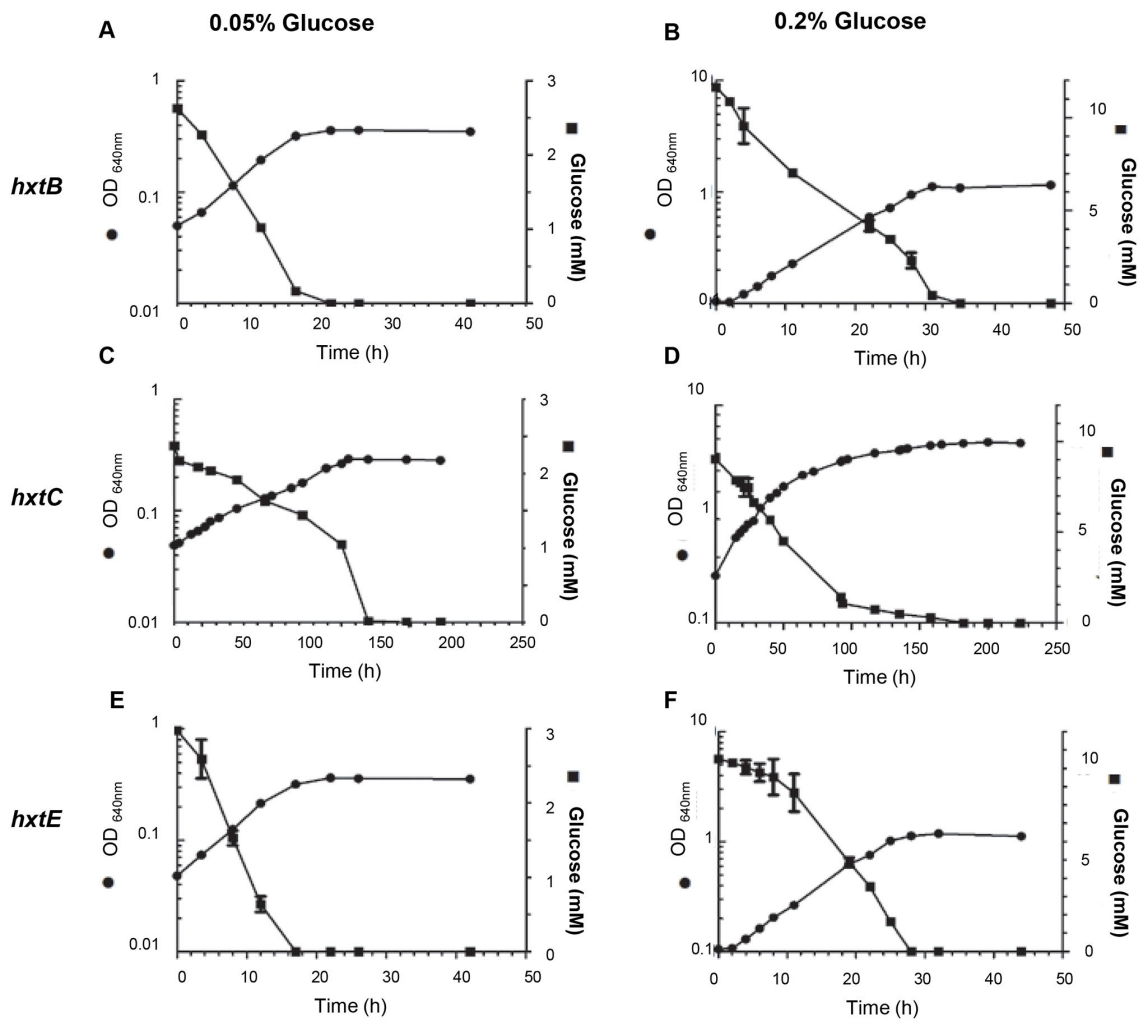
Evaluation of the growth rate, glucose consumption and kinetic parameters for *S. cerevisiae* cells (strain EBY.VW4000) expressing the *hxtB* (A and B) *–C* (C and D), and –*E* genes (E and F) grown on either 0.05 or 0.2 % glucose.

Subsequently, we concentrated our attention on the growth rate and glucose consumption of the HxtB, HxtC, and HxtE strains in YNB medium with 0.05 or 0.2% (w/v) glucose, during shake-flask aerobic batch cultivations. The S. *cerevisiae* strain expressing *hxtD* was excluded due to the absence of growth on glucose*. S. cerevisiae* expressing the *hxtB* or *hxtE* genes demonstrated highest growth rates at the glucose concentrations evaluated (Figure 6A, Table 2). The strain expressing the *hxtC* gene grew very slowly at 0.05 and 0.2 % (w/v) glucose (Figure 6B, Table 2) and no growth improvement was observed at higher glucose concentration (2%, w/v; data not shown). These findings were confirmed by the glucose consumption profile (Figures 6A-B). In addition, no ethanol production was detected in any of the glucose concentrations tested, for any of the strains expressing *hxtB, hxtC* or *hxtE* (data not shown).

**Table 2:** Specific growth rates (μ; h^-1^) of *S. cerevisiae* EBY.VW4000 strain expressing either *hxtB*, *hxtC* or *hxtE* genes grown in YNB medium with glucose at 0.05% or 0.2% (w/v) as the only carbon source.

	μ (h^-1^)	
	0.05%	0.2%
*hxtB*	0.118 ± 0.001	0.084 ± 0.002
*hxtC*	0.019 ± 0.002	0.016 ± 0.001
*hxtE*	0.110 ± 0.0002	0.098 ± 0.0011

**Figure 7:**
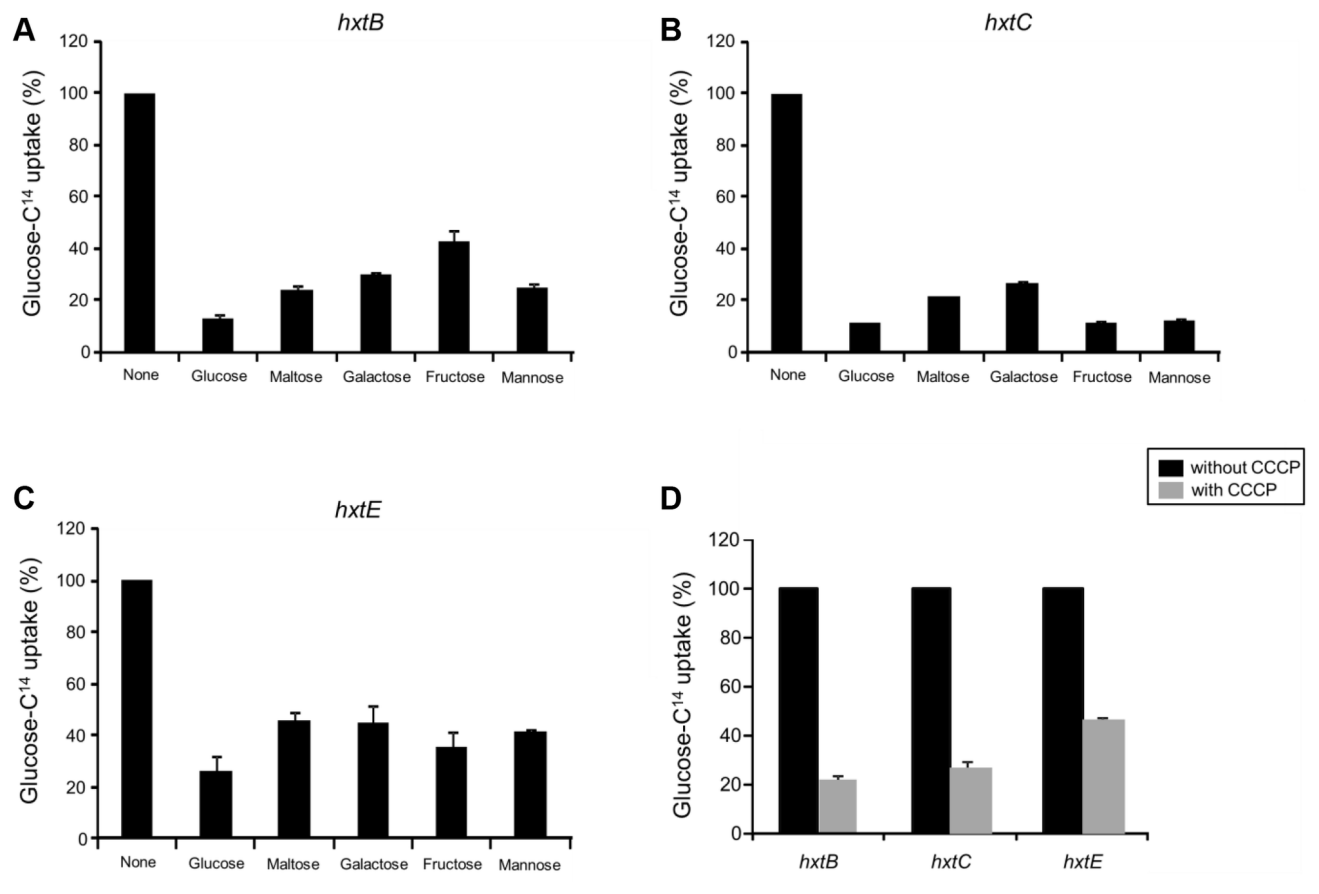
Substrate specificities of the indicated Hxt transporters. Substrate specificities of HxtB (A), HxtC (B), and HxtE (C) were determined in *S. cerevisiae* cells (strain EBY.VW4000) expressing the respective cDNA. Relative transport levels were determined in the absence of a competitor or in the presence of a tenfold excess of unlabeled glucose or a tenfold excess of unlabeled maltose, galactose, fructose, or mannose (n=3, ±, standard deviation). The results are expressed as the percentage of inhibition of the transport of radiolabelled glucose. (D) Sensitivities of the HxtB-E transporters to the uncoupler CCCP in the absence or presence of 250 μM CCCP (n=3, ±, standard deviation).

The HxtB, -C, and –E transporters were also able to accept other sugars as substrates (Figure 4). Thus, to confirm this physiological data, we studied the uptake of [^14^C]glucose in the absence or presence of either fructose, mannose or galactose as potential transport competitors (Figure 7A-C). As expected, a 10-fold excess of unlabeled glucose drastically inhibited the transport of radiolabeled glucose in the S. *cerevisiae* cells expressing *hxtB,-C*, and –*E* (Figures 7A-C). A tenfold excess of unlabeled maltose, galactose, fructose and mannose were also able to inhibit to different levels (50 to 80 %) of radiolabeled glucose transport in the S. *cerevisiae* cells expressing *hxtB,-C*, and –*E* (Figures 7A-C). These results suggest that HxtB, -C, and –E have different substrate affinities. 

To determine whether the mechanism by which HxtB, HxtC and HxtE transport glucose was by passive facilitated diffusion or active proton symport, we evaluated the sensitivity of each transporter to cyanide-*m*-chlorophenylhydrazone (CCCP), an uncoupler of transmembrane proton gradients. Upon the addition of CCCP, [^14^C]glucose uptake was affected in *S. cerevisiae* cells expressing *hxtB, -C*, and –*E*, demonstrating a 80, 70 and 55 % reduction in the respective strains (Figure 7D). Taken together, these data suggested that HxtB, HxtC, and HxtE mediated glucose transport via active proton symport.

### ^14^C-glucose transport in the null mutants of hxtB-E

 A. *n*idulans *hxtB-E* null alleles were generated using an *in vivo* S. cerevisiae fusion-based approach (see Materials and Methods). Several primary transformants that had homologous integration of either *pyrG* (*hxtD*) or *pyroA* (*hxtB,-C,-E*) at the *hxtB-E* loci were isolated and one of each gene was selected for further characterization. 

**Figure 8:**
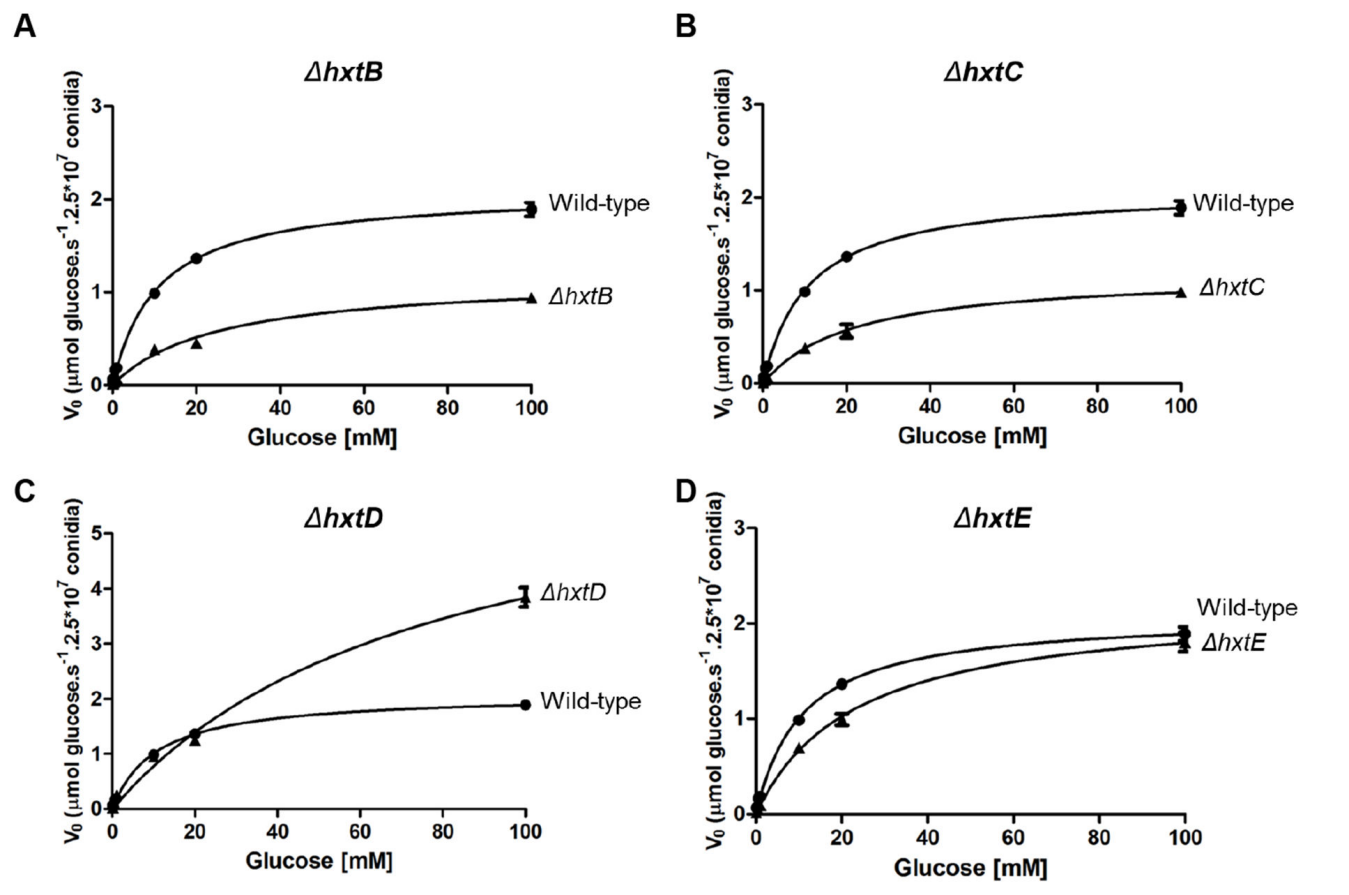
K_*m*_ values for glucose in the *A. nidulans* wild-type and *ΔhxtB-E* mutant strains. Uptake rates for [^14^C] glucose germinating conidia of the wild-type and *ΔhxtB-E* (A-D) mutant strains were determined at the indicated substrate concentrations at pH 7.0. Michaelis-Menten plots of the same data are shown (n=3, ±, standard deviation).

Since previous studies have described that glucose uptake in germinating conidia (incubated with 1.0 % glucose) is an energy dependent process [6], we evaluated the impact of each deletion on conidia germination at a this glucose concentration. In fact under these conditions, we found that glucose uptake in *A. nidulans* obeyed a single saturation kinetic with a *K*_*m*_ = 10.7 ± 0.9 mM and a *V*_max_ = 2.1 ± 0.1 µmol of glucose h^–1^ per 2.5 × 10^7^ conidia (Figure 8). The *ΔhxtB* mutant strain showed both a decreased affinity for glucose (*K*_*m*_ = 25.3 ± 3.4 mm) and a reduction in transport capacity (*V*_max_ = 1.16 ± 0.06 µmol of glucose per hour per 2.5 × 10^7^ conidia; Figure 8A). The same behaviour was also observed for *ΔhxtC* mutant strain that showed both a decreased affinity for glucose and speed of transport compared to the wild-type strain (a *K*_*m*_ = 26.0 ± 2.7 mm and a *V*_max_ = 1.20 ± 0.05 µmol of glucose per hour per 2.5 × 10^7^ conidia (Figure 8B). Interestingly, in the case of the *ΔhxtD* mutant, we also found alterations in the glucose uptake system, but this time an increase in *K*_*m*_ and *V*_max_ values to 77.6 ± 12.1 µm and 6.1 ± 0.5 µmol of glucose per hour per 2.5 × 10^7^ conidia (Figure 8C). Despite the fact that the introduction of HxtD to the EBY.VW4000S strain did not restore growth on glucose, its deletion in *A. nidulans* resulted in the loss of both glucose affinity and transport speed. The deletion of HxtE in *A. nidulans* resulted in a decrease in glucose affinity, but had little impact on the speed of transport (Figure 8D; *K*_*m*_ = 23.1 ± 2.4 mm and a *V*_max_ = 2.2 ± 0.1 µmol of glucose per hour per 2.5 × 10^7^ conidia) 

Taking into consideration the impact of each HxtB-E deletions on glucose uptake, we evaluated the growth of the null hxtB-E mutants compared to the wild-type strain on solid MM supplemented with a single carbon sources, such as glucose, xylose, maltose, glycerol, mannose, fructose, acetate, rhamnose, casein, carboxymethylcellulose, inulin, guar, peptone, and pectin at 30, 37, and 44 °C. The four strains showed the same growth and conidiation as the wild-type strain under all the tested conditions (data not shown). 

**Figure 9:**
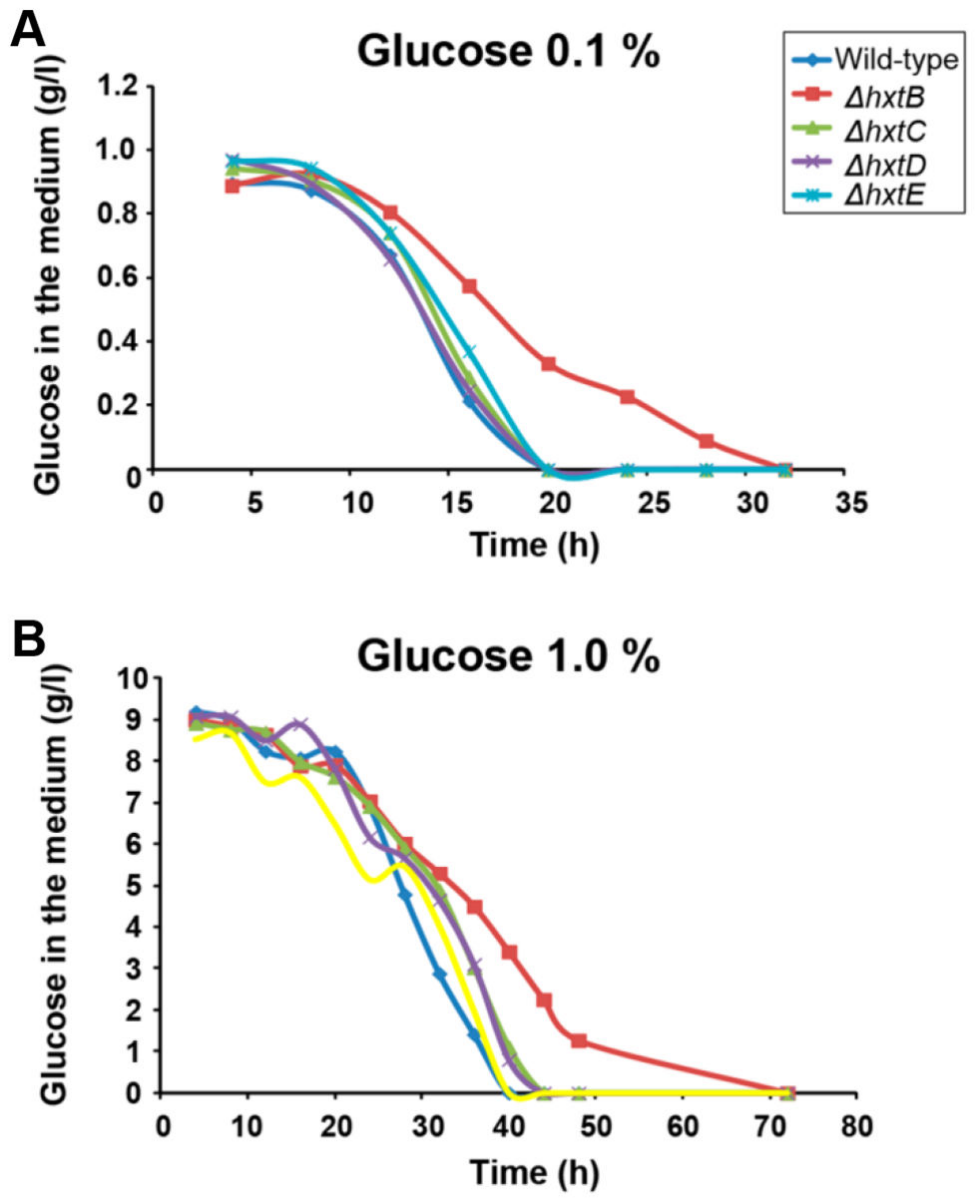
The speed of glucose consumption during growth of the wild-type and *ΔhxtB-E* *A. nidulans* mutant strains in different glucose concentrations. The wild-type and mutant strains were grown in MM+0.1 % glucose (A) or MM+1.0 % glucose and the residual glucose concentration (g/l) was determined.

Finally, we investigated the glucose consumption by growing the wild-type and the *ΔhxtB-E* in liquid MM medium with either 0.1 % or 1% of glucose (Figure 9). When grown in MM+0.1 % glucose, all the strains showed a comparable rate in glucose consumption, except for the *ΔhxtB* mutant strain which showed a delayed consumption of glucose (Figure 9A). The same behaviour was observed for the *ΔhxtB* mutant strain in MM+1.0 % glucose (Figure 9B). The lower affinity for glucose in the *ΔhxtB* mutant strain was emphasized by its increased resistance to carbon catabolite repression, when the wild-type and the mutant strains were grown in increasing concentrations of xylose+2 mM 2-deoxyglucose (2DG), which is a toxic glucose analogue (Figure S2). Taken together, these results suggest that the lack of *hxtB* results in the less efficient transport of glucose under low concentration. 

## Discussion

 Understanding how filamentous fungi can transport and sense glucose is of a topic of substantial interest to industrial mycology. As a preliminary step to identify genes involved in these processes within *A. nidulans*, we characterized four genes that showed homology to other functionally characterized fungal glucose transporters. These four genes named *hxtB-E* are from the sugar porter subfamily of the MFS transporters. Despite *hxtB-E* demonstrating high identity with the S. cerevisiae glucose sensors, Snf3p and Rgt2p (data not shown), the absence of an extended cytosolic tail, essential for the intracellular signaling role in *S. cerevisiae* [15,16,20], suggests that the *A. nidulans* proteins were transporters. However, HxtB-E possessed an extended cytosolic region within the center of the respective proteins, which could play a signaling role. Rgt2 and Snf3 have an approximately 50 amino acids long central region, while HxtB-E have 91, 129, 88, and 99 amino acids long central regions, respectively. The lack of a glutamine-rich region that acts as a mediator of protein-protein interaction, indicative of a signaling molecule, such as within the central cytosolic region of in RCO3 glucose sensor from *N. crassa*, implies otherwise [33,48]. Subsequently, biochemical and molecular assays enabled the classification of these Hxt proteins as glucose transporters. No transporters with extended cytosolic regions at either the N- or C-terminus were found in any *Aspergilli* whose genomes are available (data not shown). Thus, it is possible as suggested [49,50] that the global expression of transporters during the *A. nidulans* isotropic growth phase, i.e., during spore germination [43,49–51] might operate as a general system for sensing solute availability.

The previously characterized high affinity glucose transporter HxtA was shown to be transcriptionally induced under glucose starvation and sexual development [42]. In contrast, *hxtB-E* showed decreased mRNA accumulation during sexual development. In *A. nidulans*, *hxtB*, *-C*, *-D*, and -*E* also demonstrated increased mRNA accumulation when exposed to low glucose concentrations. The hexose transport-deficient *S. cerevisiae* strain (EBY.VW4000) has been an important tool for characterizing new hexose transporters of other fungi, such as four transporters from the hemibiotrophic plant pathogen *Colletotrichum graminicola* (CgHXT2, CgHXT3, CgHXT4 and CgHXT5) [40] and TBHXT1 transporter from the ascomycete *Tuber borchii* [36]. Subsequently, the ability of HxtB, -C, and –E to complement the growth defect of this strain on glucose, galactose, fructose, mannose, and sucrose, confirmed these proteins to be hexose transporters, while competition experiments showed them to possess a higher affinity for glucose. In contrast, HxtD was unable to restore growth, despite being localized to the S. *cerevisiae* cell membrane, on any of the tested sugar sources. It is possible glucose transport by HxtD may involve transporter cooperation with other transporter proteins interacting with each other to produce specific phenotypes aiming to achieve a high rate of glucose influx [52]. Evidence of such transport cooperation has already been demonstrated when coexpressing *Candida intermedia GXS1* glucose/xylose symporter and *GXF1* glucose/xylose facilitator [53].

Few *Aspergilli* glucose transporters that have been functionally characterized [6,41-43]. As previously shown, D-Glucose uptake in germinating wild-type *A. nidulans* conidia is an energy-requiring process mediated by transport systems with differing affinities for glucose [6]: a low-affinity system (km ~ 1.4 mM), and intermediate-affinity system (Km~400mM), and a high-affinity system (Km ~ 16 mM). To investigate the involvement of *hxtB-E* in the glucose transport system and metabolism in *A. nidulans* we generated and characterized the corresponding null mutants. We were not able to see any relevant phenotypic differences in these mutants when compared to the wild-type strain, except for the *ΔhxtB* mutant strain that showed a decreased rate of glucose consumption at low concentrations and an increased resistance to 2-DG. This indicates that although *A. nidulans* possesses other transporters capable of compensating for the absence of these transporters, the absence of HxtB has a measurable effect on glucose metabolism at low concentration. We detected a reduction on the glucose uptake for *ΔhxtB-E* mutants, with the loss of at least twice (*ΔhxtB, ΔhxtC*, and *ΔhxtE*) and seven-fold (*ΔhxtD*) affinity for glucose. Glucose uptake experiments using the S. *cerevisiae* strains expressing *hxtB*, *-C*, and –*E*, performed in the presence of CCCP, which blocks transmembrane proton gradients, strongly indicated that these *A. nidulans* transporters also act as energy-dependent glucose/H+ symporters. Many other glucose transporters, identified in filamentous fungi, such as *U. fabae HXT1* [35], glomeromycotan *GpMST1* [37], *Glomus MST2* [54], and four transporters *CgHXT1-4* from *C. graminicola* [40] have all been shown to be energy-dependent. 

The presented study set out to improve the understanding of glucose metabolism in *A. nidulans* via studying the role of four possible glucose transporters. The data described provides a clear molecular and biochemical characterization of four genes involved with glucose uptake in *A. nidulans*. 

## Materials and Methods

### Strains, media and culture methods

The genetic backgrounds of the *A. nidulans* strains used in this study are described in Table 3. Two basic types of media were used, i.e. complete and minimal. Three variants of complete media were used: YAG (2% w/v glucose, 0.5% w/v yeast extract, 2% w/v agar, trace elements), YUU (YAG supplemented with 1.2 g/liter [each] of uracil and uridine), and liquid YG or YG+UU medium with the same composition but without agar. A modified minimal media (original high-nitrate salts, trace elements, 2% w/v agar, pH 6.5) containing either 2%, 1%, 0.1% glucose (w/v) or no carbon source were used. Trace elements, vitamins, and nitrate salts were included as described by 55. *A. nidulans* strains were grown at 37°C unless indicated otherwise. 

**Table 3:** Plasmids and *A. nidulans* and *S. cerevisiae* strains used in this work.

**Plasmids/Strains**	**Genotype**	**Reference**
pRS426	*amp* ^*R*^ * lacZ* URA3	[63,]
pCDA21	*Zeo::pyr ampR*	[64]
pRH195 *	pBluescript II SK+, TRP1, CEN6, ARSH4+ P_HXT7_-XKS1-T_HXT7_	[65]
TNO2A3	*pyroA4 pyrG89; chaA1*; Δ*nKuA::argB*	[60]
*ΔhxtB*	*pyroA4 pyrG89; chaA1*; Δ*nKuA::argB; ΔhxtB::pyroA4*	This work
*ΔhxtC*	*pyroA4 pyrG89; chaA1*; Δ*nKuA::argB; ΔhxtC::pyroA4*	This work
*ΔhxtD*	*pyroA4 pyrG89; chaA1*; Δ*nKuA::argB; ΔhxtD::pyrG*	This work
*ΔhxtE*	*pyroA4 pyrG89; chaA1*; Δ*nKuA::argB; ΔhxtE::pyroA4*	This work
SC9721	MATa his 3-D200 URA 3-52 leu2D1 lys 2D202 trp 1D63	FGSC
EBY.VW4000	MATK leu2-3,112 ura3-52 trp1-289 his3-v1 MAL2-8c SUC2 hxt17v hxt13v : :loxP hxt15v: :loxP hxt16v: :loxP hxt14v : :loxP hxt12v: :loxP hxt9v: :loxP hxt11v: :loxP hxt10v: :loxP hxt8v : :loxP hxt514v: :loxP hxt2v: :loxP hxt367v : :loxP gal2v stl1v : :loxP agt1v : :loxP ydl247wv: :loxP yjr160cv: :loxP	[47]

* The original vector pRH195 carries the XKS1 gene which was released after digestion with Spe*I* and Sal*I*. The resultant vector without the XKS1 gene was used in this work for compl ementation assays.

The S. *cerevisiae* sugar transporter knockout strain EBY.VW4000 (*CEN.PK2-1C Δhxt1-17 Δstl1 Δagt1 Δydl247w Δyjr160c Δgal2*) [45] was used for the *in vivo* complementation phenotype assays. The S. *cerevisiae* SC9721 strain (*MATα his3-Δ200 URA3-52 leu2Δ1 lys2Δ202 trp1Δ63*) acquired from the Fungal Genetic Stock Center (FGSC) was used for *in vivo* recombination. Yeast strains were cultivated at 30°C in synthetic medium (SC, 0.67% Difco yeast nitrogen base without amino acids, 0.083 % amino acid drop out mix) supplemented with glucose or another specific carbon source.

### Construction of *A. nidulans hxtB-E* null mutants

Standard genetic techniques for *A. nidulans* were used for all strain constructions and genetic transformation [55,56]. DNA manipulations were performed according to [57]. All PCR reactions were performed using Phusion High-Fidelity DNA polymerase (New England Biolabs), except for the amplification of whole cassettes where *TaKaRa Ex Taq DNA Polymerase* (Clontech USA) was used. All the primers used in this work are listed in Table S1.

Deletion cassettes for *ΔhxtB, C* and *E* (AN6669, AN10891 and AN1797, respectively) were constructed by *in vivo* recombination in *S. cerevisiae* as previously described [58]. Briefly, a construct consisting of a 1.0-kb region of the 5´-UTR and 3´-UTRs (primers P1-6 and P7-12 respectively) flanking each of the target genes and the *A. fumigatus pyroA* gene (P13 and P14; used as a selective marker for pyridoxine prototrophy) was constructed by *in vivo* recombination in *S. cerevisiae*. The 5´-UTR, 3´-UTR and *pyroA* fragments plus the linearized pRS426 vector cut with *Eco*RI and *Bam*HI, were purified from agarose gel and transformed into *S. cerevisiae* SC9721 strain using the lithium acetate method [59]. The external 5´-UTR Forward and 3´-UTR Reverse primers possessed cohesive ends with the vector pRS426 and the internal primers 5´-UTR R and 3´-UTR F contained cohesive ends with 5´ and 3´sequence of *pyro* gene. All cassettes were PCR-amplified from genomic DNA extracted from the respective *S. cerevisiae* transformant, purified and used to transform *A. nidulans* strain TNO2a3 (*ΔnkuA*) strain [60], according to [56]. Transformants were scored for their ability to grow on minimal medium without pyridoxine and homologous integration confirmed by PCR (Figure S1). The deletion cassette for *ΔhxtD* was acquired from the FGSC. This cassette carried the *pyrG* gene as a selective marker for uridine and uracil prototrophy. The deletion cassette was PCR amplified using specific primers (P15 and P16) 

*S. cerevisiae* genomic DNA was extracted by using the protocol described by 61. All cassettes were PCR-amplified using *TaKaRa Ex Taq DNA Polymerase* (Clontech) and used for transformation of wild-type *A. nidulans* strain TNO2a3 (*ΔnkuA*) strain [60] according to [56]. Transformants were scored for their ability to grow on minimal medium without uridine and uracil and checked by PCR to confirm their homologue integration. 

### RNA extraction and Real-time PCR reactions

Asexual spore development was synchronized by transferring a thin mycelial mat, filtered from liquid culture, to an agar plate. To induce sexual development, we incubated the mycelia for 11 days (0–2 days: conidiophore development and asexual development; 2–11 days: cleistothecia development and sexual development; 6–11 days: presence of ascospores). Mycelia were harvested, washed twice with dH_2_O and immediately frozen in liquid nitrogen. The mycelia were then lyophilized, disrupted by grinding in liquid nitrogen and total RNA was extracted using the RNeasy Plant Mini Kit (Qiagen). To check RNA integrity, 10 µg of RNA was fractionated in 2.2 M formaldehyde, 1.2% agarose gel, stained with ethidium bromide, and visualized under UV-light. A total of 20 µg of RNA were treated with RNAse-free DNAse (Promega), purified with RNeasy Mini Kit (Qiagen) and then quantified on a NanoDrop 2000 Thermo Scientific). The SuperScript III First Strand Synthesis system (Invitrogen) and oligo(dT) primers were used for cDNA synthesis, according to the manufacturer’s protocol. All RT-qPCR reactions were performed using an ABI 7500 Fast Real-Time PCR System (Applied Biosystems) and Taq-Man™ Universal PCR Master Mix kit (Applied Biosystems). The RT-qPCR reactions and calculations were performed according to [62]. The primers and Lux™ fluorescent probes (Invitrogen) used in this work are described in Table S1.

### Constructions for *S. cerevisiae* complementation assays

The sugar transporter deletion strain EBY.VW4000 was used for the S. *cerevisiae* complementation assays [45]. More than 20 sugar transporters and sensors including *HXT1-17* and *GAL2* have been deleted from this strain [45]. For this reason, the strain is unable to grow on D-glucose, but it can grow on maltose, as a single carbon source. The hxtB-E ORFs were PCR amplified from *A. nidulans* cDNA using specific primers P29-30, P31-32, P33-34 and P35-36, respectively (Table S1). Note that the reverse primers included the stop codon. The forward and reverse primers (P29-36) possessed cohesive ends for the modified vector pRH195 (under the control of the *HXT7* promoter and terminator) which was double digested with *Spe*I and *Sal*I to liberate the *XKS1* gene and linearize the vector. The purified linearized plasmid and PCR-amplified sugar transporter ORFs were transformed into *S. cerevisiae* EBY.VW4000 strain by lithium acetate method [59], where they underwent *in vivo* recombination. Transformants were selected for tryptophan prototrophy on a SC medium supplemented with tryptophan and 2% maltose (SC-Trp). Genomic DNA of single colonies was isolated as described by 62 and the specific ORFs were PCR amplified using specific primers. Single transformed colonies were analyzed for their ability to grow on SC-Trp medium supplemented with either 2% glucose or 0.2% glucose.

The subcellular localization of hxtB-D in *S. cerevisiae* was checked by constructing hxtB-E::GFP cassettes. Thus, these ORFs were tagged with GFP at their C-terminal. The GFP gene was separated from the target ORF by the Spacer-GFP [63,]. Briefly, each ORF were PCR amplified from cDNA of the *A. nidulans* A4 strain using primers P29 and 37 (hxtB), P31 and 38 (hxtC), P33 and 39 (hxtD) and finally P35 and 40 (hxtE) (Table S1). The forward primers included the Spacer-GFP sequence and omitted the stop codon. The forward and reverse primers possessed cohesive ends with the vector modified pRH195, which was double digested with *Spe*I and *Sal*I for linearization. The GFP gene containing the stop codon was amplified from pMCB17apx (kindly provided by Vladimir P. Efimov; primers P41 and P42) (Table S1) and the forward primer possessed cohesive ends with the modified vector pRH195 which was double digested with *Spe*I and *Sal*I for linearization. In order to get *in vivo* recombination in *S. cerevisiae*, the linearized modified pRH195 plasmid was purified from agarose gel and transformed into *S. cerevisiae* EBY.VW4000 strain with PCR-amplified sugar transporter ORFs and GFP gene by lithium acetate method [59]. The transformants were selected for tryptophan prototrophy on a SC medium supplemented with tryptophan and 2% maltose (SC-Trp). Genomic DNA of single colonies was isolated as described by 59 and the specific ORFs were PCR amplified using specific primers. Single transformed colonies were analyzed for their ability to grow on SC-Trp medium supplemented with either 2% glucose or 0.2% glucose.

### Liquid growth conditions for *S. cerevisiae*

*S. cerevisiae* EBY.VW4000 expressing hxt-B, -C, -D and -E were grown in YNB medium supplemented with different carbon sources. The cultures were performed in flasks containing a 2:1 ratio of gas to liquid phase in an orbital shaker (160 rpm) at 26°C. Growth was monitored via OD measurements at 640 nm, while aliquots were taken at each time point to evaluate the concentration of glucose and ethanol in the medium.

### Estimation of glucose and ethanol concentrations using *S. cerevisiae* strains

Glucose and ethanol concentrations in the media were assayed by high-performance liquid chromatography, using a Refractive Index detector and a HyperREZ XP Organic Acids (8µm 100mm x 7.70mm) column at 57°C. The column was eluted with 2.5 mM of sulphuric acid at a flow rate of 0.7 ml/min. The sample was injected through Gilson 234 auto-injector, with a retention time for glucose of 7.43 min and for ethanol of 15.29 min.

### Estimation of cell dry weight for *S. cerevisiae*

The dry weight of *S. cerevisiae* cells (DW) from the different transformants was determined using pre-weighed aluminium caps. After removal of the medium by centrifugation, the cellular samples were washed with 4 volumes of ice-cold dH_2_O and transferred to the aluminium caps for drying overnight at 80°C before being reweighed. Parallel samples varied by less than 1%.

### Evaluation of free glucose in the extracellular culture medium

 For the glucose uptake assay, a total of 1x 10^7^ spores were inoculated in 100 ml of MM containing 1% or 0.1% glucose, maintained at 37°C in an orbital shaker. Aliquots (3 ml) of the supernatant were collected after 4, 8, 12, 16, 20, 24, and 48 hours and stored at -20°C. The enzymatic kit Glucose GOD-PAP Liquid Stable Mono-reagent (LaborLab Laboratories Ltda) was used to measure free glucose in the medium, according to the manufacturer’s specifications.

### *A. nidulans* glucose uptake assay

Glucose uptake rates were measured by assaying the incorporation of D-[U-^14^C] glucose [289.0 mCi/mmol (10.693 GBq)/mmol] (Perkin Elmer Life Sciences) in germinating conidia at various D-glucose concentration according to [6] with modifications. Briefly, 1.2 x 10^9^ conidia were inoculated into 600 ml MM containing 1% D-glucose (w/v) as a carbon source. Incubation was carried out for 6 h at 37°C in an orbital shaker at 180 rpm. Germinating conidia were harvested by filtration over nitrocellulose filters (Fisherbrand) mounted in a vacuum manifold and washed twice with ice-cold water to eliminate traces of glucose. For glucose transport analysis, aliquots of 250 μl (of 2.5 x 10^7^ germinating conidia) containing D-glucose [0.1-100mM] were dispensed into 2 ml tubes plus 1 μl of radiolabelled ^14^C-glucose (0.2 μCi) and incubated at 37°C. After incubation for 30 to 60 seconds, uptake was immediately quenched by the addition of 1.5 ml ice-cold water and filtration over nitrocellulose filters (Fisherbrand) mounted in a vacuum manifold, followed by two consecutive washes with 1.5 ml of ice-cold water. Filters were subsequently transferred to 8 ml of ScintiSafeTM Econo1 scintillation liquid (Fisher Scientific). The D-[U-14C] glucose taken up by cells was measured using Tri-Carb® 2100TR Liquid Scintillation Counter. 

### CCCP assays

For CCCP (carbonylcyanide *m*-chlorophenylhydrazone) assays using *S. cerevisiae* strains, 500 ml of SC-Trp medium supplemented with 0.2 % glucose was incubated at 30°C with EBY.WV4000 strain harboring one of the *hxtB*, *hxtC* or *hxtE* genes. Cultures started from an initial OD_640_ 0.1 and were grown until reached OD_640_ ~ 0.6. Cells were harvested by centrifugation (4000 rpm), washed twice with 50 ml ice-cold water and resuspended in 1.250 ml of water. A total of 400 μl of cells was diluted in 800 μl of water and aliquots of 40 μl incubated at 30°C for 5 min to allow temperature equilibration. Subsequently, 10 μl of water containing 250 μM of CCCP were added 5 minutes before or concomitantly with 0.2 μCi of ^14^C-glucose. Subsequently, the reaction was immediately stopped by quenching with 1.5 ml ice-cold water and filtration over nitrocellulose filters (Fisherbrand) mounted in a vacuum manifold, followed by two consecutive washes with 1.5 mL of ice-cold water. Filters were subsequently transferred to 8 ml of ScintiSafeTM Econo1 scintillation liquid (Fisher Scientific). The D-[U-14C] glucose taken up by cells was measured using Tri-Carb® 2100TR Liquid Scintillation Counter. 

## Supporting Information

Figure S1 **PCR confirmation of homologue integrations for *A. nidulans* mutants *ΔhxtB, ΔhxtC, ΔhxtD* and *ΔhxtE*.**
(TIF)

Figure S2 **Growth phenotypes of *A. nidulans* wild-type and *ΔhxtB-E* mutants grown on different concentrations of xylose (**A**) or xylose plus 0.2 mM 2-deoxy-glucose (2-DG).**
(TIF)

Table S1 **Primers and probes used in this work.**
(DOC)
