# Supplementary figures and images for: Identification of Glucose Transporters in Aspergillus nidulans
Source: PLoS One. 2013 Nov 25;8(11):e81412. doi: 10.1371/journal.pone.0081412 (PMC3839997; doi:10.1371/journal.pone.0081412)

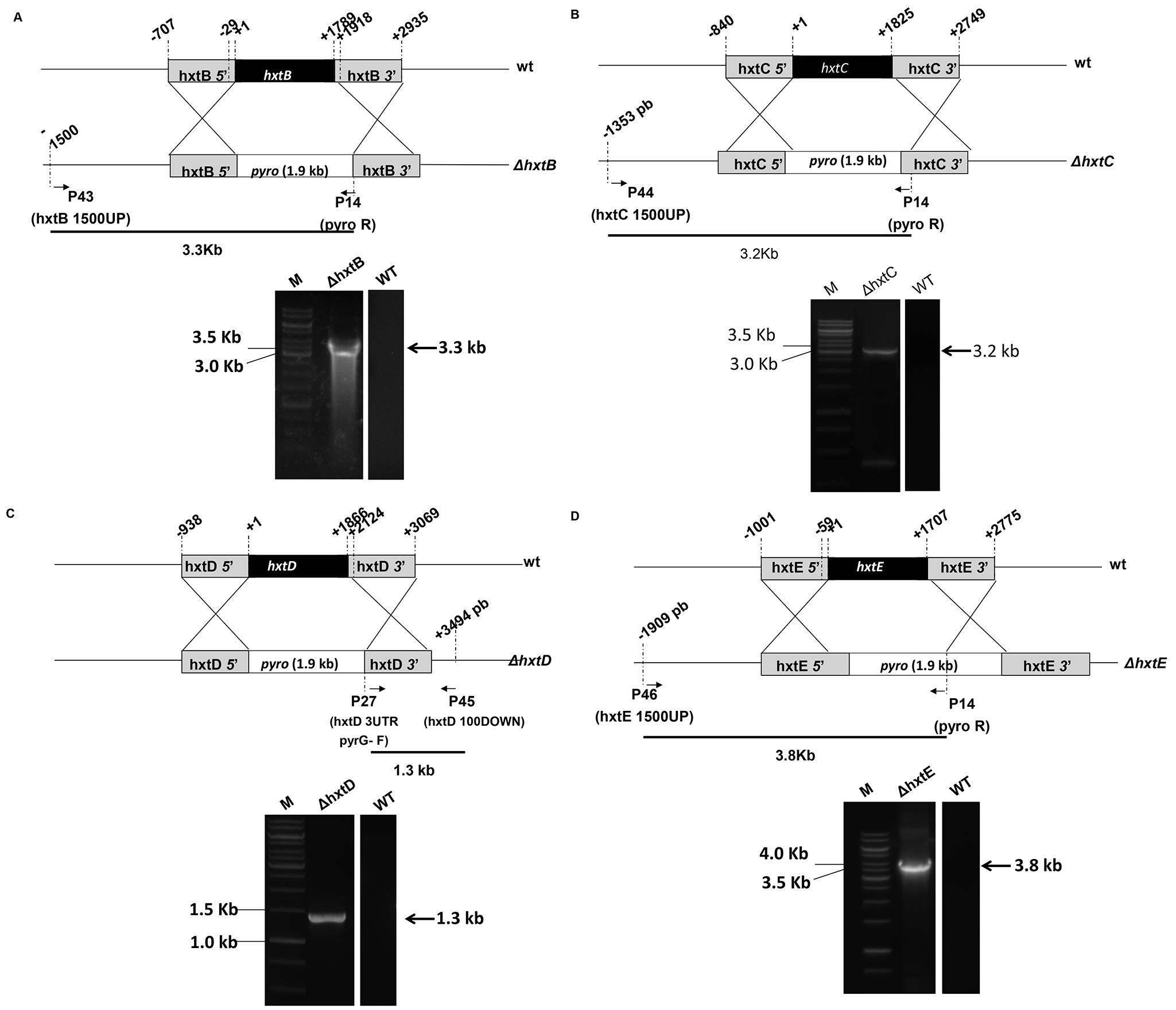

Supplement: Figure S1 — PCR confirmation of homologue integrations for A. nidulans mutants ΔhxtB, ΔhxtC, ΔhxtD and ΔhxtE. (TIF) [file pone.0081412.s001.tif]

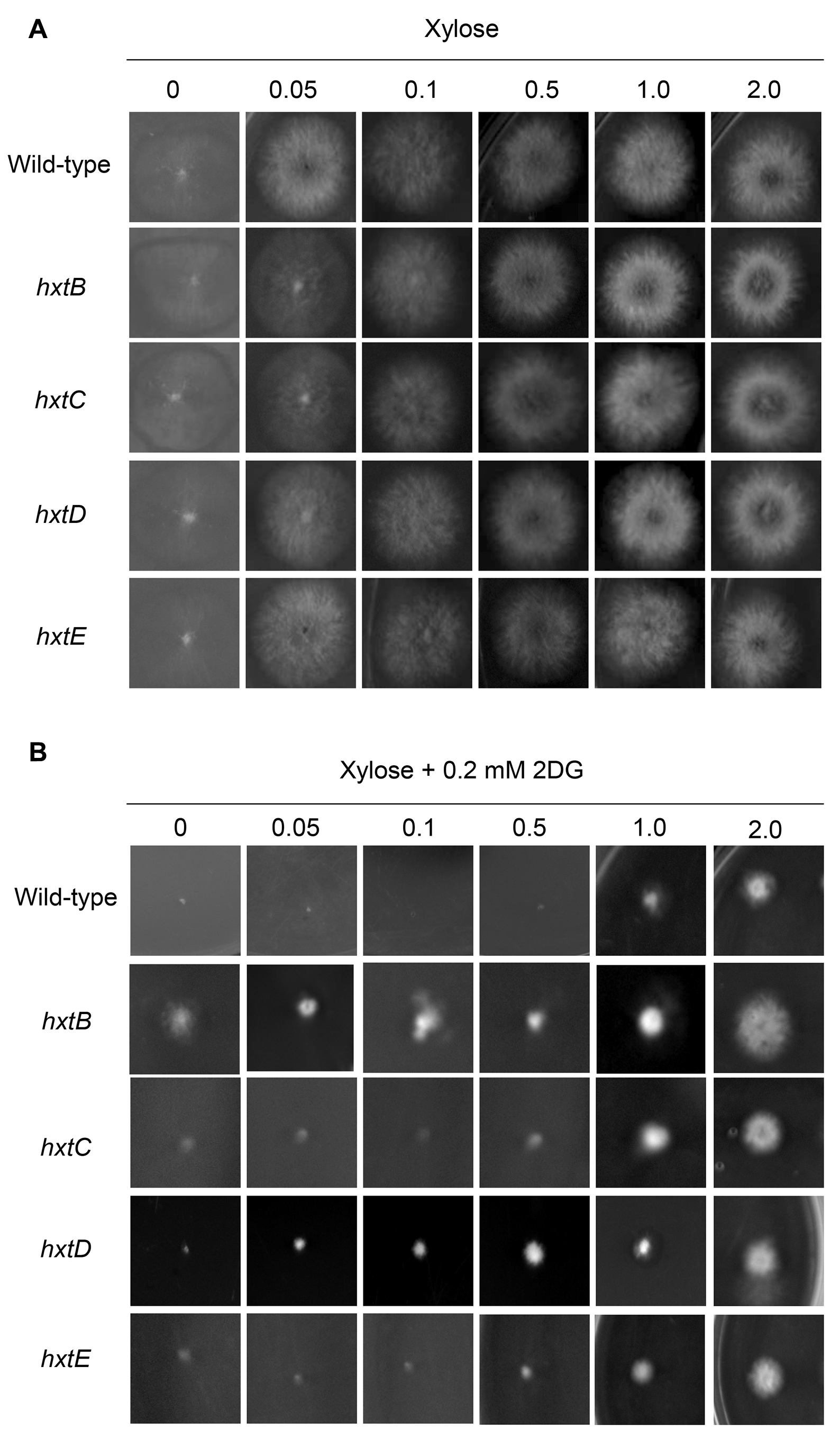

Supplement: Figure S2 — Growth phenotypes of A. nidulans wild-type and ΔhxtB-E mutants grown on different concentrations of xylose (A) or xylose plus 0.2 mM 2-deoxy-glucose (2-DG). (TIF) [file pone.0081412.s002.tif]
