# Supplementary material for: Identification of Glucose Transporters in Aspergillus nidulans
Source: PLoS One. 2013 Nov 25;8(11):e81412. doi: 10.1371/journal.pone.0081412 (PMC3839997; doi:10.1371/journal.pone.0081412)
Supplement: Table S1 — Primers and probes used in this work. (DOC) [file pone.0081412.s003.doc]

Supplementary Table S1 - Primers and probes used in this work

|  | **Primer name** | **5´-3´sequence** |
| --- | --- | --- |
| P1 | hxtB 5UTR tag pRS426- F | GTAACGCCAGGGTTTTCCCAGTCACGACGCCGTCTTAGCCTGTCTTAGT |
| P2 | hxtB 5UTR tag pyro- R | GACCCAACAACCATGATACCAGAGGAAAGTGGGAGTGAAG |
| P3 | hxtC 5UTR tag pRS426- F | GTAACGCCAGGGTTTTCCCAGTCACGACGCCAGACCTCAGGGCCAAGAAAAGC |
| P4 | hxtC 5UTR tag pyro- R | GACCCAACAACCATGATACCAGGTTTTATGTCGATCGCAGAATGGC |
| P5 | hxtE 5UTR tag pRS426- F | GTAACGCCAGGGTTTTCCCAGTCACGACGGGAAGTGAGACGCTTTTTGGACG |
| P6 | hxtE 5UTR tag pyro- R | GACCCAACAACCATGATACCAGGAGGGGAAGCAAAACAAGACG |
| P7 | hxtB 3UTR tag pRS426 R | GCGGTTAACAATTTCTCTCTGGAAACAGCGTAGGATGAGTAGGCAAGA |
| P8 | hxtB 3UTR tag pyro- F | CTGTCGATCATGTGGATGCTTACAGGCTTCATGTCGTTGG |
| P9 | hxtC 3UTR tag pRS426 R | GCGGTTAACAATTTCTCTCTGGAAACAGCGGAGTACGACGCATGGTCTTGTTACG |
| P10 | hxtC 3UTR tag pyro- F | CTGTCGATCATGTGGATGCTTGGTCAGCGAAGGTGGATCC |
| P11 | hxtE 3UTR tag pRS426 R | GCGGTTAACAATTTCTCTCTGGAAACAGCCCTTTTTCGCAGGCCATTCTGG |
| P12 | hxtE 3UTR tag pyro- F | CTGTCGATCATGTGGATGCTATCTGCCCACGTCAGCCTTCTCTC |
| P13 | pyro F | TGGTATCATGGTTGTTGGGTC |
| P14 | pyro R | AGCATCCACATGATCGACAG |
| P15 | hxtD 5UTR tag pRS426- F | GTAACGCCAGGGTTTTCCCAGTCACGACGGTATACACGACAGACTCGGA |
| P16 | hxtD 3UTR tag pRS426- R | GCGGATAACAATTTCACACAGGAAACAGCGGCCGTTACTGACGTACTAT |
| P17 | hxtB_spacer GFP-R | AGTTCTTCTCCTTTACTCATTCCCCGTGTTCCCGCCTTCTTAGCAATGTCC |
| P18 | hxtC_spacer GFP-R | AGTTCTTCTCCTTTACTCATTCCCCGTGTTCCGACCGACACCCCATCAG |
| P19 | hxtD_spacer GFP-R | AGTTCTTCTCCTTTACTCATTCCCCGTGTTCCGGCAGCGCTTTCCACGGTTG |
| P20 | hxtE_spacer GFP-R | AGTTCTTCTCCTTTACTCATTCCCCGTGTTCCGACCGAAGCTTTCTCGACATGC |
| P21 | pyrG tag GFP- F | GCATGCAAGCTTGGCGTATTCTGTCTGAGAGGAGGC |
| P22 | pyrG-R | GAATTCGCCTCAAACAATGCTCTTCACC |
| P23 | Spacer GFP- F | GGAACACGGGGAATGAGTAAAGGAGAAGAACTTTTCACTGG |
| P24 | GFP VE3’AF- R | CTCAGACAGAATACGCCAAGCTTGCATGC |
| P25 | hxtB 3UTR tag pyrG- F | GCATTGTTTGAGGCGAATTCGGAATACAGCAAGGATGTCCC |
| P26 | hxtC 3UTR tag pyrG- F | GCATTGTTTGAGGCGAATTCTGGTCAGCGAAGGTGGATCC |
| P27 | hxtD 3UTR tag pyrG- F | GCATTGTTTGAGGCGAATTCGGAATACAGCAAGGATGTCCC |
| P28 | hxtE 3UTR tag pyrG- F | GCATTGTTTGAGGCGAATTCGGAATACAGCAAGGATGTCCC |
| P29 | hxtB pRH195 -R | GTTTTTTTAATTTTAATCAAAATGGCGGACGGTGTCG |
| P30 | hxtB pRH195 -F | GAATTAATAAAAGTGTTCGCTTAATCCTTCTCAGTCATACCCA |
| P31 | hxtC pRH195 -R | GTTTTTTTAATTTTAATCAAAATGGGCATTCTCTTCAAAAAGC |
| P32 | hxtC pRH195 -F | GAATTAATAAAAGTGTTCGCCTAGACCGACACCCCATCAGG |
| P33 | hxtD pRH195 -R | GTTTTTTTAATTTTAATCAAAATGGCAGACGCGGTGATTG |
| P34 | hxtD pRH195 -F | GAATTAATAAAAGTGTTCGCTTAGGCAGCGCTTTCCAC |
| P35 | hxtE pRH195 -R | GTTTTTTTAATTTTAATCAAAATGGGCTTTATGCTTAGG |
| P36 | hxtE pRH195 -F | GAATTAATAAAAGTGTTCGCCTACTCGACATGCTCCGA |
| P37 | hxtB pRH195 spacerGFP-F | AGTTCTTCTCCTTTACTCATTCCCCGTGTTCCATCCTTCTCAGTCATACCCA |
| P38 | hxtC pRH195 spacerGFP-F | AGTTCTTCTCCTTTACTCATTCCCCGTGTTCCGACCGACACCCCATCAGG |
| P39 | hxtD pRH195 spacerGFP-F | AGTTCTTCTCCTTTACTCATTCCCCGTGTTCCGGCAGCGCTTTCCACGGTTG |
| P40 | hxtE pRH195 spacerGFP-F | AGTTCTTCTCCTTTACTCATTCCCCGTGTTCCCTCGACATGCTCCGA |
| P41 | pRH195 GFP-F | GAATTAATAAAAGTGTTCGCTTAACGCCAAGCTTGCATGC |
| P42 | pRH195 spacerGFP- R | GGAACACGGGGAATGAGTAAAGGAGAAGAACTTTTCACTGG |
| P43 | hxtB 1500UP | GTCTTGTGACGGCTTGTTTCTGCC |
| P44 | hxtC 1500UP | CCAGACCTCAGGGCCAAGAAAAGC |
| P45 | hxtD 100DOWN | TCTCCCGAGCAGTGGCCGA |
| P46 | hxtE 1500UP | GTACAGAGTAGACCTGCAAGTGC |
| P47 | *tubC* | GCAGAATGTCTCGTCCGAATG  CACTTTATGCCGTCGCCGAAAG[FAM]G |
| P48 | *hxtA* | TTCGCCCTCGGATATGCTT  CGGAAATCGCCTCAGCTATGGTTC[FAM]G |
| P49 | *hxtB* (AN6669) | GGTCTCCTCCATCATCTTGTCG  CGGGCTACACCTACTTCCTGATTCC[FAM]G |
| P50 | *hxtC* (AN10891) | GGACTTGCCCAGGCTGACTT  CGGATCAAGACCACGAGTCCATC[FAM]G |
| P51 | *hxtD* (AN8737) | CAGAGGAGTTGGAGGGCAATC  CGGTTGAACTATGGGACGGAGAAC[FAM]G |
| P52 | *hxtE* (AN1797) | ACGCAGACGAAAGCGATAAGG  CGGTGTATTGTCGGCACAACCAC[FAM]G |
| P53 | nsdD Primer | CCTCCTGGTCGATGCCATAGT |
| P54 | nsdD Sonda | CGGCAATTTCGCGTAGTGTAAGC[FAM]G |
